# The effect of a combined intervention on exclusive breastfeeding in primiparas: A randomised controlled trial

**DOI:** 10.1111/mcn.12948

**Published:** 2020-01-13

**Authors:** Drita Puharić, Mario Malički, Josip Anđelo Borovac, Vladimir Šparac, Boris Poljak, Nađa Aračić, Nero Marinović, Nives Luetić, Irena Zakarija‐Grković

**Affiliations:** ^1^ Postgraduate Doctoral Program “TRIBE,” School of Medicine University of Split Split Croatia; ^2^ Department of Research in Biomedicine and Health, Department of Medical Humanities, School of Medicine University of Split Split Croatia; ^3^ Department of Pathophysiology, School of Medicine University of Split Split Croatia; ^4^ University Hospital of Split Split Croatia; ^5^ Medical Centre “Šparac” Split Croatia; ^6^ “Cito” Medical Centre Split Croatia; ^7^ Split‐Dalmatiani County Health Department Omiš Croatia; ^8^ Split‐Dalmatian County Health Department Split Croatia; ^9^ Departments of Clinical Skills and Family Medicine, School of Medicine University of Split Split Croatia

**Keywords:** antenatal/postnatal intervention, breastfeeding, exclusive breastfeeding, first‐time mothers, proactive telephone support, RCT

## Abstract

An antenatal/postnatal intervention involving proactive telephone support and written materials was conducted among primiparas. Four hundred women, from the Split‐Dalmatia County, Croatia, were randomized between November 2013 and December 2016 into three groups: intervention (IG), active control (ACG) and standard care (SCG). Primary outcome was exclusive breastfeeding (EBF) at 3 months. Secondary outcomes included breastfeeding difficulties, attitudes towards infant feeding, breastfeeding self‐efficacy and social support. Practice staff were blinded to group allocation. Of 400 women, 45 (11%) were lost to follow‐up, and final analyses were conducted on 129 (IG), 103 (ACG) and 123 (SCG) participants. EBF rates at 3 months were significantly higher for the IG (odds ratio [OR] 4.6, 95% confidence interval [CI], 2.7 to 8.1; EBF 81%) as well as at 6 months (OR 15.7, 95% CI, 9.1 to 27.1; EBF 64%) compared with SCG (EBF 47% at 3 months and 3% at 6 months). Higher rates were also observed for the ACG at 3 months (OR 2.2, 95% CI, 1.3 to 3.8, EBF 68%) and 6 months (OR 2.3, 95% CI, 1.4 to 3.9, EBF 16%). Participants in the IG had the highest increase in positive attitudes towards infant feeding, in comparison to baseline, and significantly higher breastfeeding self‐efficacy. Participants in SCG experienced significantly more breastfeeding difficulties, both at 3 and 6 months, in comparison to AC and IGs. Written breastfeeding materials and proactive telephone support among primiparas are an effective means of increasing breastfeeding rates, decreasing breastfeeding difficulties and improving self‐efficacy and attitudes towards infant feeding.

**Key messages**

The provision of a combined antenatal and postnatal intervention, involving proactive telephone support and a breastfeeding booklet, among first time mothers:

- increases exclusive breastfeeding at 3 and 6 months
- increases breastfeeding self‐efficacy
- improves attitudes towards infant feeding
- decreases the occurrence of breastfeeding difficulties

## 1 INTRODUCTION

Breastfeeding is associated with numerous established health benefits for both the infant and mother (Victora et al., 2016) as well as considerable potential savings to health services (Rollins et al., 2016). These outcomes are even greater for infants that are exclusively breastfed. Hence, the World Health Organisation (WHO) recommends exclusive breastfeeding (EBF) for the first 6 months of an infants' life followed by ongoing breastfeeding, along with timely and appropriate complementary foods, for at least 2 years (World Health Organisation [WHO], 2001).

Despite this, EBF rates in Croatia are far from recommended. A cohort study conducted in 2011 found that 96% of women initiated breastfeeding, of which 39% were EBF at 3 months, and only 8% were still practicing this form of infant nutrition at 6 months (Zakarija‐Grkovic, Boban, Janković, Ćuže, & Burmaz, 2017) Maintaining breastfeeding, especially EBF, is challenging for many new mothers. Women need continuous support from skilled individuals. Numerous intervention studies have been conducted to try and improve breastfeeding rates. A 2017 Cochrane review of 73 studies involving 74,656 mother–infant pairs found that any form of additional breastfeeding support increased both the duration and exclusivity of breastfeeding. Several factors were found to improve EBF: interventions delivered with a face‐to‐face component, high background initiation rates of breastfeeding, lay support and a specific schedule of four to eight contacts (McFadden et al., 2017).

Telephone communication is increasingly being used as a practical and cost‐effective form of support within health care. A 2013 Cochrane review of telephone support for postpartum women (Lavender, Richens, Milan, Smyth, & Dowswell, 2013) identified eight intervention trials aiming at improving breastfeeding outcomes at 6 weeks, 3 and 6 months. Results were inconsistent for any or EBF at 6 weeks, whereas at 3 and 6 months, women receiving telephone support were more likely to be EBF.

Based on these findings, we aimed to test (a) the effect of an educational intervention in the form of a breastfeeding booklet distributed during pregnancy and (b) the effect of four proactive telephone calls provided by a health professional during the prenatal and postnatal period, on EBF rates at 3 and 6 months among first time mothers in a setting with high initiation rates. In addition, we assessed the effect of these interventions on “any breastfeeding” rates and breastfeeding difficulties at 3 and 6 months, as well as breastfeeding self‐efficacy, infant feeding attitudes and social support at 3 months.

## 2 METHODS

The full study protocol can be found online (Zakarija‐Grković, Puharić, Malički, & Hoddinott, 2016). A brief overview, in accordance with CONSORT (Moher et al., 2012) reporting guidelines, is shown here.

### 2.1 Trial design

This was a single‐centre, controlled, randomized, three‐arm, superiority study, with blind‐outcome assessment.

### 2.2 Setting

The Split‐Dalmatia County, with a population of 452,841, is serviced by two maternity hospitals: a tertiary referral hospital in the administrative centre, Split, with 4,188 births in 2017 (27.3% caesarean rate), and a small local hospital in the town of Sinj, where 53 uncomplicated deliveries took place in the same year. Eight obstetric practices were included in this study – of which half were private – where women were recruited by nursing and medical staff, during routine visits, between November 2013 and December 2016.

### 2.3 Study participants' eligibility criteria

The study population was primigravidae, with a singleton pregnancy, who attended their primary care obstetrician between 20 to 32 weeks of pregnancy. In addition, they were required to speak Croatian and reside within the territory of the Republic of Croatia for at least a year. Those who were unable to communicate in Croatian by phone, planning to leave the country within a year or had a severe medical or psychiatric problem that could be aggravated by participating in the study, were excluded.

### 2.4 Interventions

Patients were randomly assigned to one of three groups:

- Intervention group (IG) – received a breastfeeding booklet and a general, pregnancy booklet, followed by four proactive telephone calls – one in pregnancy and three after delivery, at 2, 6 and 10 weeks.
- Active control group (ACG) – received a general, pregnancy booklet, followed by four proactive telephone calls – one in pregnancy and three after delivery, at 2, 6 and 10 weeks.
- Standard care group (SCG) – received standard care, that is, did not receive any written materials or phone calls before or after birth.

The intervention in this study consisted of printed educational materials and four proactive phone calls. The breastfeeding booklet contained information from Session three (“Promote breastfeeding during pregnancy”) of the United Nations Children's Fund (UNICEF)/WHO “Baby‐friendly Hospital Initiative” 20‐hr course for maternity staff (United Nations Children's Fund [UNICEF] & WHO, 2006). Topics included the importance of EBF, skin‐to‐skin contact, colostrum, correct attachment (with illustrations), rooming‐in, feeding on demand, breastfeeding after 6 months with the introduction of other foods, how to recognize if the baby is getting enough milk and the risks of not breastfeeding, including costs and environmental impact. Apart from the breastfeeding booklet, mothers in the IG also received a general, pregnancy booklet (obtained from a parenting website, with permission), as did the mothers in the ACG. The breastfeeding booklet had four pages in colour and 10 images, whereas the general, pregnancy booklet had four pages in colour and eight images. Telephone support aimed to provide women with relevant information, support and encouragement, using Michie's behaviour change technique (Michie et al., 2013). All interventions were conducted by DP, a registered nurse with 15 years of clinical experience, of which 2 years were spent working in a primary care obstetric practice, and who completed additional breastfeeding training.

### 2.5 Outcome measures

The primary outcome of this study was the proportion of mothers EBF at 3 months, measured using a postal, self‐completed infant feeding survey. In addition, we measured the proportion of EBF infants at 6 months, so as to enable comparison with other studies. Secondary outcomes included any breastfeeding, prevalence of childhood illnesses (mother‐reported), prevalence of breastfeeding difficulties and whether help was sought, maternal BMI, infant weight gain and reasons for stopping breastfeeding at 3 and 6 months, whereas attitudes towards infant feeding, breastfeeding self‐efficacy and social support were measured at 3 months only. In our study, “EBF” refers to infants who received breast milk only, as well as those who received water in addition to breast milk (known as “predominant breastfeeding,” EU, 2008). We combined them into one group given the known similar clinical outcomes for these infants and the small sample size of the latter group in our study (*n*=11). Disaggregated data are available in Table A2. Attitudes were measured using the Iowa Infant Feeding Attitude Scale (IIFAS; De La Mora, Russell, Dungy, Losch, & Dusdieker, 1999, Marinović Guić, 2007). The breastfeeding self‐efficacy scale (Pavicic Bosnjak, Rumboldt, Stanojevic, & Dennis, 2012) was used to estimate women's confidence in breastfeeding. A Croatian version of the Social Support Appraisal Scale—SS‐A was used to evaluate how much women felt supported by friends and family (Hudek‐Knežević, 1994; Vaux et al., 1986). Questions about breastfeeding difficulties were included in the questionnaire on infant feeding practices. Participants were recruited from November 2013 till December 2016, with latest outcome measures collected in May, 2017.

### 2.6 Sample size

A priori power analysis, published in our protocol, was calculated based on previously published breastfeeding rate of 34% in Croatia (Zakarija‐Grković et al., 2012) and an expected (minimal rate) of 50% for the IG (power 0.80, and alpha at 0.05) with 5% estimated drop‐out rate. Target planned sample size was 459; however, recruitment stopped at 400 invited participants due to time constraints of the lead researcher.

### 2.7 Randomization and blinding

Two obstetric practices were added atop those planned in the protocol, to aid recruitment, bringing the total to eight. Practice staff recruited eligible women to the study and, once consented, forwarded their details to the lead investigator who randomized each participant to one of three arms of the study, using a computer random number list pregenerated by a member of the research team. Stratified randomization was performed based on known predictors of EBF, namely, smoking status (no, yes or stopped during pregnancy) and educational level (elementary school, high school or college), to ensure balance in each group. Practice staff were blinded to group allocation, and study participants were not told of the existence and differences between the study groups; they were only informed that a study on infant feeding was being held.

### 2.8 Statistical analysis

Intention to treat analysis was conducted using the “worst‐case” scenario: all initially randomized participants in the IG were considered to have stopped breastfeeding, and those in the active control and SCGs as if they continued with EBF. As the primary outcome analysis (breastfeeding rates and associated odds ratios) in intention to treat analysis showed the same direction of results as analysis based only on the patients that were fully followed (Table A1), all primary and secondary analyses below are reported on the participants that were fully followed. Additionally, although we did not per protocol plan to collect data on predominant breastfeeding, participants provided information for it, so we also present those results in Table A2.

Main outcome data (the rates of breastfeeding), as well as participants' nominal data, are reported using the number and percentages calculated based on total number of participants in each group, and the differences between the groups tested using chi‐square tests and ordinal regression for the primary outcome (standard group serving as a comparison). Numerical outcomes of secondary outcomes and participant characteristics due to their nonnormal distribution were reported as medians and interquartile ranges, and group differences were tested with Kruskall‐Wallis test. Possible association of participants' characteristics and the type of breastfeeding at 3 and 6 months was tested using ordinal regression analyses. For the regression analyses categorical variables were dichotomized wherever possible (exception being education which we grouped into primary or secondary school, college and university).

### 2.9 Evaluation of telephone records

As per protocol, a 10% (*n* = 40) random sample of phone calls was selected and assessed for fidelity by a trained psychologist, independent from the research team. The lead investigator was found to consistently adhere to the study protocol, applying a tailored person‐centred approach.

## 3 RESULTS

**Figure 1:**
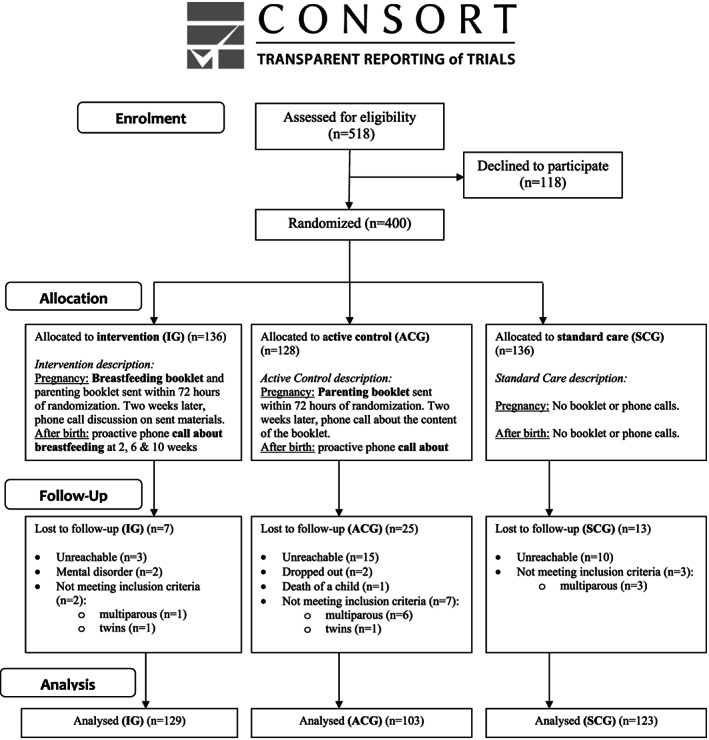
Study flow diagram

Of the 518 eligible women approached during their antenatal visit, 118 declined to participate, with the rest randomized to one of three groups. One‐hundred and thirty‐six women were allocated to the IG, 128 to the ACG and 136 to the SCG. All participants who underwent randomization (*n* = 400) received the intervention as allocated. Recruitment through eight practices lead to 12 (3%) ineligible participants being included, which, upon discovery, were thanked and their participation was discontinued. An additional 33 (8%) were lost to follow‐up, primarily because they could not be contacted (Figure 1), resulting in 129, 103 and 123 participant data being analysed in the IG, ACG and SCG, respectively.

Baseline data of participants, collected during pregnancy, are presented in Table 1. Fourteen percent of participants identified as smokers, and almost all participants (99%) had completed at least 12 years of formal education. Almost half of the participants (48%) were unsure about future pacifier use, unlike intention to breastfeed, where almost all participants stated that they intended to feed their infants either exclusively or partially with breast milk (61% and 38%, respectively). A large majority (87%) of participants planned to exclusively breastfeed at least 4–6 months, with almost two thirds of women (64%) planning to breastfeed more than 6 months.

**Table 1:** Participants' baseline data (*n*=355)

Variable (n, %)	Intervention (n=129)	Active control (n=103)	Standard care (n=123)
**Smoking**			
No	85 (66)	58 (56)	78 (63)
Yes	17 (13)	14 (14)	18 (15)
Stopped in pregnancy	27 (21)	31 (30)	27 (22)
**Education**			
Primary	2 (2)	0 (0)	0 (0)
Secondary	61 (47)	42 (41)	55 (45)
College	20 (16)	21 (20)	18 (15)
University	46 (36)	40 (39)	50 (41)
**BMI (Md, IQR)**	24.5 (22.2‐26.1)	24.1 (21.9‐26.8)	24.8 (23.1‐26.9)
**Pacifier use intention**			
No	22 (17)	8 (8)	13 (11)
Yes	54 (43)	37 (36)	48 (39)
Don't know	51 (40)	58 (56)	61 (50)
**Employment**			
Employed	103 (80)	74 (72)	89 (72)
Unemployed	25 (19)	26 (25)	30 (24)
Other source of income	1 (1)	3 (3)	4 (3)
**Economic sector**			
Primary	0 (0)	0 (0)	0 (0)
Secondary	86 (68)	67 (66)	84 (69)
Tertiary	36 (28)	26 (25)	27 (22)
Other (i.e. housewife, student)	5 (4)	9 (9)	10 (8)
**Father's employment**			
Employed	113 (88)	91 (88)	110 (89)
Unemployed	8 (6)	7 (7)	10 (8)
Other source of income	8 (6)	5 (5)	3 (2)
**Partner status**			
Lives with partner	127 (98)	102 (99)	123 (100)
Single	2 (2)	1 (1)	0 (0)
**House ownership**			
Owns flat	84 (65)	72 (70)	86 (70)
Rented flat	26 (20)	15 (15)	23 (19)
Lives with parents	17 (13)	13 (13)	14 (11)
Other	3 (2)	3 (3)	0 (0)
**No. of household members**			
1	1 (1)	1 (1)	3 (2)
2	90 (70)	69 (67)	84 (68)
3	15 (12)	15 (15)	11 (9)
4	14 (11)	13 (13)	15 (12)
5	7 (5)	3 (3)	8 (7)
6	1 (1)	1 (1)	2 (2)
7	1 (1)	1 (1)	0 (0)
**Monthly income (EUR)**			
< 472	34 (26)	33 (32)	37 (30)
472‐950	95 (74)	70 (68)	84 (68)
>950	0 (0)	0 (0)	2 (2)
**Age**			
<18	1 (1)	2 (2)	4 (3)
18 – 24	30 (23)	21 (20)	29 (24)
25 – 35	91 (71)	72 (70)	73 (59)
>35	6 (5)	8 (8)	17 (14)
**Breastfeeding intention**			
No BF	1 (1)	1 (1)	0 (0)
EBF	85 (66)	57 (55)	73 (60)
Mixed feeding	43 (33)	45 (44)	48 (40)
**Intended duration of EBF**			
< 1mo.	2 (2)	1 (1)	5 (4)
1 – 3 mo.	9 (7)	12 (12)	10 (8)
4 – 6 mo.	116 (91)	89 (87)	104 (87)
**Intended duration of any BF**			
1 – 3 mo.	3 (3)	3 (3)	3 (3)
4 – 6 mo.	33 (28)	28 (29)	30 (26)
7 – 12 mo.	50 (43)	47 (48)	65 (57)
13 – 18 mo.	20 (17)	15 (15)	12 (10)
19 – 24 mo.	7 (6)	4 (4)	4 (3)
>24 mo.	4 (3)	1 (1)	1 (1)

* Chi‐squared tests for all variables except BMI which was compared with Kruskal‐Wallis tests.

EBF – Exclusive Breastfeeding

BF – Breastfeeding

Primary outcome measure, that is, the proportion of women exclusively breastfeeding (EBF) at 3 months, showed significant differences between the groups, with the highest rate found in the IG (*n*=105, 81%), followed by ACG (*n*=70, 68%) and SCG (*n*=58, 47%). At 6 months the EBF rates were 64% (*n*=82) vs 16% (*n*=16) vs 3% (*n*=4) in the IG, ACG and SCG, respectively (Table 2). Compared with standard care (SCG), general pregnancy support (SCG) was associated with a slight increase in EBF at 3 months (odds ratio [OR] 2.2, 95% confidence interval [CI], 1.3 to 3.8), and written breastfeeding support (IG) with a significant increase (OR 4.6, 95% CI, 2.7 to 8.1). At 6 months, the association for SCG remained similar (OR 2.3, 95% CI, 1.4 to 3.9), and for the IG it greatly increased (OR 15.7, 95% CI, 9.1 to 27.1).

**Table 2:** Participants' breastfeeding (BF) practice at 3 and 6 months postpartum

Breastfeeding practice (n, %)	Intervention (n=129)	Active control (n=103)	Standard care (n=123)
**At 3 months**			
Exclusive BF	105 (81)	70 (68)	58 (47)
Any BF	10 (8)	13 (13)	25 (20)
No BF	14 (11)	20 (19)	40 (33)
Odds Ratio (95% CI)*	4.6 (2.7 to 8.1)	2.2 (1.3 to 3.8)	reference category
**At 6 months**			
Exclusive BF	82 (64)	16 (16)	4 (3)
Any BF	25 (19)	47 (46)	46 (37)
No BF	22 (17)	40 (39)	73 (59)
Odds Ratio (95% CI)*	15.7 (9.1 to 27.1)	2.3 (1.4 to 3.9)	reference category

* Ordinal Regression

Secondary outcome measures per group are presented in Tables 3, 4, 5. The IG had a slightly better attitude towards infant feeding (as measured by IIFAS scale) at baseline and had the highest increase in those attitudes, as well as significantly higher breastfeeding self‐efficacy compared with the SCG (Table 3). Both during the first and the second 3‐month period, participants in the SCG experienced the highest number of breastfeeding difficulties, mainly insufficient milk, and during the first 3 months, more experienced illness of a child.

**Table 3:** Participants' attitudes towards infant feeding, social support and breastfeeding self‐efficacy

Scale (Md, IQR)	Intervention (n=129)	Active control (n=103)	Standard care (n=123)	P*
**IIFAS**	n=127	n=101	n=103	
Baseline	60 (60‐68)	57 (48‐64)	57 (49‐64)	**0.037**
3 months	70 (65‐74)	66 (61‐71)	62 (56‐69)	**<0.001**
**CSS‐A**	n=127	n=101	n=103	
Baseline	94 (89‐95)	94 (91‐96)	93 (90‐95)	0.387
3 months	96 (92‐96)	94 (91‐96)	95 (91‐96)	0.739
**BSES**	n=113	n=79	n=78	
3 months	65 (57‐70)	63 (54‐68)	59 (53‐66)	**0.001**

* Kruskal‐Wallis test

Scale acronyms: IIFAS ‐ Infant feeding attitudes, CSS‐A ‐ Croatian version of Social Support scale, SS‐A BSES ‐ Breastfeeding Self‐Efficacy.

**Table 4:** Participants' experiences during the first 3 months postpartum

**Variable (n, %)**	**Intervention (n=129)**	**Active control (n=103)**	**Standard care (n=123)**	**P** *
**Childhood illness**				
No	120 (93)	86 (83)	93 (76)	**0.005**
Yes	9 (7)	16 (16)	29 (24)	
Missing data	0 (0)	1 (1)	1 (1)	
**Medical assistance**				
No	1 (11)	0 (0)	1 (3)	0.367
Yes	8 (89)	16 (100)	28 (97)	
Missing data	0 (0)	0 (0)	0 (0)	
**Trouble breastfeeding**				
No	54 (42)	42 (41)	33 (27)	**0.025**
Yes	75 (58)	61 (59)	90 (73)	
**No. of breastfeeding difficulties (Md, IQR)**	2 (1‐2)	2 (1‐3)	2 (1‐3)	0.752
**Asked for breastfeeding help**				
No	5 (4)	9 (9)	9 (7)	0.293
Yes	124 (96)	94 (91)	114 (93)	
**No of different help interventions (Md, IQR)**	4 (3‐4)	3 (2‐4)	3 (2‐4)	**0.009**
**Self‐reported reasons for stopping breastfeeding**	n=10	n=13	n=26	
Insufficient milk	5 (50)	3 (23)	11 (42)	0.117
Caesarean section	0 (0)	2 (15)	5 (19)	
Child breast rejection	0 (0)	2 (15)	4 (15)	
Mother's illness	1 (10)	2 (15)	0 (0)	
Stinging breast	0 (0)	0 (0)	1 (4)	
Mastitis	1 (10)	0 (0)	2 (8)	
Bad experience during hospital stay	2 (20)	0 (0)	0 (0)	
Other	1 (10)	4 (31)	3 (12)	
**BMI at 3 months (Md, IQR)**	23.2 (21.8‐25.3)	23.7 (21.4‐25.4)	24.0 (21.4‐26.5)	0.933
**BMI before pregnancy (Md, IQR)**	21.5 (20.2‐22.9)	21.3 (19.7‐24.1)	21.6 (20.2‐23.0)	0.955
**Child weight progress in kg (Md, IQR)**				
1^st^ month	1.0 (0.8‐1.1)	1.0 (0.9‐1.1)	1.0 (0.8‐1.2)	0.296
2^nd^ month	1.0 (0.9‐1.1)	1.0 (0.8‐1.1)	1.0 (0.8‐1.0)	0.679
3^rd^ month	1.0 (0.8‐1.0)	1.0 (0.8‐1.0)	1.0 (0.8‐1.0)	0.605

* Chi‐squared tests for all variables except BMI, No. of breastfeeding difficulties, and No. of different help interventions which were compared with Kruskal‐Wallis tests.

**Table 5:** Participants experiences during the 3–6 months postpartum

Variable (n, %)	Intervention (n=129)	Active control (n=103)	Standard care (n=123)	P*
**Childhood illness**				
No	118 (91)	89 (86)	104 (85)	0.530
Yes	9 (7)	11 (11)	16 (13)	
Missing data	2 (2)	3 (3)	3 (2)	
**Medical assistance**				
No	2 (22)	0 (0)	0 (0)	0.042
Yes	7 (78)	11 (100)	16 (100)	
**Trouble breastfeeding**				
No	117 (91)	81 (79)	88 (72)	**0.002**
Yes	12 (9)	22 (21)	33 (27)	
Missing data	0 (0)	0 (0)	2 (2)	
**No. of breastfeeding difficulties (Md, IQR)**	1 (1‐2)	1 (1‐2)	1 (1‐1)	0.098
**Asked for breastfeeding help**				
No	89 (69)	69 (67)	78 (63)	0.188
Yes	40 (31)	34 (33)	42 (34)	
Missing data	0 (0)	0 (0)	3 (2)	
**No of different help interventions (Md, IQR)**	2 (1‐3)	2 (2‐3)	2 (1‐3)	0.428
**Reasons for stopping breastfeeding**	n=3	n=13	n=26	
Insufficient milk	1 (33)	6 (55)	7 (70)	0.393
Child breast rejection	0 (0)	2 (18)	0 (0)	
Other	2 (67)	3 (27)	3 (30)	
**BMI at 6 months (Md, IQR)**	22.3 (20.9‐24.4)	22.7 (20.2‐24.2)	23.3 (20.9‐25.7)	0.551
**Child weight progress in kg (Md, IQR)**				
4^th^ month	0.8 (0.7‐1.0)	1.0 (0.9‐1.2)	1.0 (0.9‐1.2)	0.181
5^th^ month	0.8 (0.6‐1.0)	0.8 (0.7‐1.1)	0.9 (0.7‐1.0)	0.135
6^th^ month	0.8 (0.7‐1.0)	0.9 (0.7‐1.2)	0.9 (0.7‐1.1)	0.091

* Chi‐squared tests for all variables except BMI at 6 months, No. of breastfeeding difficulties, and No. of different help interventions which were compared with Kruskal‐Wallis tests.

Adjusting the regression model for all baseline characteristics yielded ORs very similar to the unadjusted ones (reported above): IG was significantly associated with EBF at 3 months (OR 5.1, 95% CI, 2.6 to 9.7), with ACG (OR 2.6, 95% CI, 1.4 to 4.7), intention not to use a pacifier (OR 2.9, 95% CI, 1.0 to 7.9), and older (25+) age (OR 2.0, 95% CI, 1.03 to 3.7) having slightly weaker associations. At 6 months, associations were IG (OR 21.4, 95% CI, 11.1 to 41.2), and ACG (OR 2.5, 95% CI, 1.4 to 4.4), with initial intention to breastfeed up to 6 months having a negative association (OR 0.1, 95% CI, 0.02 to 0.9).

In the first 3 months postpartum most participants relied on breastfeeding help from community nurses (*n*=241, 68%), their relatives (*n*=237, 67%) and friends (*n*=171, 48%, Table A3), with their relatives and friends continuing to be the biggest help in the 3‐ to 6‐month period (Table A4).

## 4 DISCUSSION

This study shows that written breastfeeding information and telephone support during pregnancy and at 2, 6 and 10 weeks postpartum leads to a significant increase in EBF rates among first time mothers. Additionally, mothers who received nonbreastfeeding‐focused written information and telephone support showed a slight increase in EBF rates compared with the SCG, which is in line with McFadden's systematic review finding of any support leading to increased breastfeeding rates. In addition, participants who received the breastfeeding‐focused intervention had a significantly more positive attitude toward infant feeding and higher breastfeeding self‐efficacy at three months postpartum, compared with control groups, as well as significantly fewer breastfeeding difficulties during the first 6‐month period.

Our positive results may have been achieved partly due to the type of written materials provided to participants during the study. Firstly, plain language was used in all written materials and pretested by a pilot sample of 40 expectant women. Secondly, the pregnancy booklet was written by a Croatian parenting organization, that is, for parents, by parents. In addition, the breastfeeding booklet used evidence‐based information from the chapter “Promoting breastfeeding during pregnancy,” part of the UNICEF/WHO 20‐hr course for maternity staff. Behaviour change technique intervention components, such as “health consequences,” “social and environmental consequences” and “emotional consequences” were also highlighted as part of the antenatal written information (Michie et al., 2013) An easy‐to‐read A4 format was chosen, with plenty of illustrations, and sent to participants' home addresses, enabling equal access by all to the information provided. Educational materials for expectant couples, provided free of charge and free of conflict of interest, may be especially useful in settings where antenatal course attendance rates are low, such as in the City of Split, where only 16% of women were found to have attended an antenatal course (Zakarija‐Grković et al., 2017).

A key aspect of the intervention was the telephone support, provided, proactively, by a trained health professional, at scheduled intervals during the antenatal and postnatal period. This enabled women to predict when support would be available, providing them with a safety‐net and source of easily accessible information – most likely explaining the significantly lower incidence of breastfeeding difficulties among women in the intervention and ACGs. Of importance may be the fact that one person (DP) carried out all the phone calls with study participants, in order to ensure continuity of care and establish trust, adapting to each mother according to her needs and preferences. Similar support could be provided in everyday practice given that most mothers are under the care of a community/district/visiting nurse or midwife and telephone access is nowadays readily available and inexpensive. At the same time, motivation to provide continuous support to mothers may present a challenge outside of the study setting.

Other studies have also found improved breastfeeding rates as a result of additional breastfeeding support. In a 2016 Cochrane review of interventions for increasing the initiation of breastfeeding (Balogun et al., 2016), women who received health care professional‐led breastfeeding education and support were significantly more likely to initiate breastfeeding (average risk ratio 1.43, 95% CI, 1.07 to 1.92; Tau^2^ = 0.07, *I* ^2^ = 62%, low‐quality evidence) compared with those women who received standard care. Similarly, a 2017 updated Cochrane review (McFadden et al., 2017) based on 73 randomized controlled trials, involving 74 656 mother–infant pairs, confirmed that all forms of extra support lead to an increase in any breastfeeding, especially in settings where breastfeeding initiation rates are high, such as in Croatia. In our study, extra support in the form of proactive telephone calls led to a decrease in cessation of EBF.

Furthermore, despite initial intentions of almost all participants to exclusively breastfeed for at least 4–6 months (91% IG, 87% ACG and SCG), only 3%, 16% and 64% of participants actually exclusively breastfed at 6 months in the SCG, ACG and IG, respectively. This represents a difference of 27%, 71% and 84% in IG, ACG and SCG, respectively. These figures should be interpreted with caution given that intended duration of EBF was assessed as “for at least 4–6 months,” which is not the same as intended duration of EBF for 6 months. Still, the discrepancy between intention and realization is great, prompting us to ask ourselves why this occurred and how can we better support women to realize their breastfeeding goals. Breastfeeding is not a single woman's task – it is a collective responsibility, in which the whole community plays an important role. Obstetricians play a key role, given their ready access to expectant mothers and influence. They are ideally suited to provide educational materials to expectant couples, including conflict‐free information on breastfeeding, and yet despite this, expectant mothers in the Split‐Dalmatia County did not routinely receive any educational materials.

We did not directly ask participants whether they were satisfied with the proactive breastfeeding support they received, but indirect comments made by participants were all positive. As all of our phone support was conducted by one person, interventions aiming to replicate this support may be influenced by the skill and warmth of the persons who conduct them. It can be assumed that the research nurse was highly motivated to provide phone support to participating mothers, which is less likely to be achieved in real life (i.e., having all professionals equally motivated for providing support and ensuring continuity of support). Hence, further studies on the intervention are needed to evaluate outcomes when support is provided by other health professionals. Although we did not recruit all of the patients we planned according to our protocol, due to time constraints of the main investigator, our sample size calculation was based on data which greatly differed from the findings we observed in our study. This is most likely due to the 10‐year time lapse during which breastfeeding awareness has been influenced by numerous promotional campaigns. Our final differences were greater than predicted, allowing our sample to have sufficient power to demonstrate the effectiveness of the intervention. Initially, we also planned to collect infant birth demographics, but it proved more difficult to do so, and hence, these were omitted. Despite these limitations, methodological rigour was adhered to, resulting in comparable groups at baseline, minimal attrition, avoidance of the Hawthorne effect and fidelity in implementation of planned intervention. In addition, participants were followed‐up for a relatively long period of time, during which woman‐centred care was consistently provided in a pro‐active manner.

## 5 CONCLUSIONS

Written breastfeeding materials and proactive telephone support among first time mothers are an effective means of increasing breastfeeding rates, decreasing breastfeeding difficulties and improving breastfeeding self‐efficacy and attitudes towards infant feeding during the first six months postpartum.

## CONFLICTS OF INTEREST

The authors declare that they have no conflicts of interest.

## CONTRIBUTIONS

DP conceived the study, analysed the data and wrote the initial draft. MM analysed the data, wrote the results and revised the manuscript. IZG contributed to study design, co‐wrote the initial draft, analysed the data and revised the manuscript. JAB entered data into the database. VŠ, BP, NA, NM and NL recruited participants. All authors approved the final version of the manuscript.

## APPENDIX A

**Table A1:** Participants' breastfeeding (BF) practice 6 months postpartum based on imputed data for lost‐to follow‐up participants

Breastfeeding practice	Intervention (*n*=136)	Active control (*n*=128)	Standard care (*n*=136)
At 3 months			
Exclusive BF	105 (77)	95 (74)	71 (52)
Any BF	10 (7)	13 (10)	25 (18)
No BF	21 (15)	20 (16)	40 (29)
Odds Ratio (95% CI)^a^	5.9 (3.4 to 10.1)	1.4 (0.9 to 2.2)	reference category
At 6 months			
Exclusive BF	82 (60)	41 (32)	17 (13)
Any BF	25 (18)	47 (37)	46 (34)
No BF	29 (21)	40 (31)	73 (54)
Odds Ratio (95% CI)^a^	18.3 (10.7 to 31.2)	1.8 (1.1 to 2.9)	reference category

*Note.* Data were imputed based on the “worst‐case” scenario: all participants lost to follow‐up in active control (*n*=25), and standard care group (*n*=13) were considered to still exclusively breastfeed, and those in the intervention group (*n*=7) to not breastfeed.

a Ordinal regression.

**Table A2:** Participants' breastfeeding (BF) practice at 3 and 6 months post‐partum

Breastfeeding practice	Intervention (*n*=129)	Active control (*n*=103)	Standard care (*n*=123)
At 3 months			
Exclusive BF	102 (79)	63 (61)	56 (46)
Predominant BF	3 (2)	7 (7)	2 (2)
Any BF	10 (8)	13 (13)	25 (20)
No BF	14 (11)	20 (19)	40 (33)
At 6 months			
Exclusive BF	73 (57)	12 (12)	4 (3)
Predominant BF	9 (7)	4 (4)	0 (0)
Any BF	25 (19)	47 (46)	46 (39)
No BF	22 (17)	40 (39)	73 (59)

*Note.* Exclusive breastfeeding category was dichotomized into exclusive and predominant BF (addition of water).

**Table A3:** Type and frequency of breastfeeding assistance participants sought in the first 3 months postpartum

Type of help	Intervention (*n*=129)	Active control (*n*=103)	Standard care (*n*=123)	Total (*n*=355)
Pregnancy course	16 (12)	11 (11)	15 (12)	42 (12)
Breastfeeding support group	17 (13)	17 (17)	16 (13)	50 (14)
Counsellors from the Split Parenting Club	16 (12)	10 (10)	12 (10)	38 (11)
RODA‐SOS hotline	16 (12)	8 (8)	6 (5)	30 (8)
La Leche League leader in Split	0 (0)	0 (0)	1 (1)	1 (0)
UNICEF's hotline	3 (2)	1 (1)	7 (6)	11 (3)
Breastfeeding‐friendly practice	1 (1)	0 (0)	0 (0)	1 (0)
Community nurse	84 (65)	66 (64)	91 (74)	241 (68)
Paediatrician	45 (35)	24 (23)	46 (37)	115 (32)
Family doctor	7 (5)	5 (5)	3 (2)	15 (4)
A friend	68 (53)	46 (45)	57 (46)	171 (48)
Relatives	94 (73)	61 (59)	82 (67)	237 (67)
Is not specified	3 (2)	2 (2)	1 (1)	6 (2)
Hospital nurse	4 (3)	5 (5)	10 (8)	19 (2)
Facebook support	0 (0)	0 (0)	1 (1)	1 (5)
Intervention nurse	34 (26)	4 (4)	1 (1)	39 (11)
Myself	1 (1)	4 (4)	3 (2)	8 (2)
Internet	23 (18)	19 (18)	16 (13)	58 (16)
Gynecologist	0 (0)	1 (1)	2 (2)	3 (1)
Various literature	1 (1)	0 (0)	0 (0)	1 (0)
IBCLC	1 (1)	0 (0)	0 (0)	1 (0)
Materials provided by the intervention nurse	1 (1)	0 (0)	0 (0)	1 (0)

**Table A4:** Type and frequency of breastfeeding assistance participants sought in the 3–6 months postpartum

Type of help	Intervention (*n*=129)	Active control (*n*=103)	Standard care (*n*=123)	Total (*n*=355)
Pregnancy course	4 (3)	0 (0)	1 (1)	5 (1)
Breastfeeding support group	6 (5)	1 (1)	12 (10)	19 (5)
Moms Counsellors from the Split Parenting Club	3 (2)	0 (0)	2 (2)	5 (1)
RODA‐SOS hotline	4 (3)	1 (1)	4 (3)	9 (3)
UNICEF's hotline	2 (2)	0 (0)	2 (2)	4 (1)
Community nurse	1 (1)	3 (3)	5 (4)	9 (3)
Paediatrician	9 (7)	18 (17)	12 (10)	39 (11)
Family doctor	0 (0)	1 (1)	1 (1)	2 (1)
A friend	18 (14)	20 (19)	19 (15)	57 (16)
Relatives	27 (21)	21 (20)	26 (21)	74 (21)
Not specified	2 (2)	0 (0)	1 (1)	3(1)
Intervention nurse	3 (2)	1 (1)	0 (0)	4 (1)
Myself	0 (0)	1 (1)	2 (2)	3 (1)
Internet	9 (7)	4 (4)	4 (3)	17 (5)

Abbreviation: UNICEF: United Nations Children's Fund.
